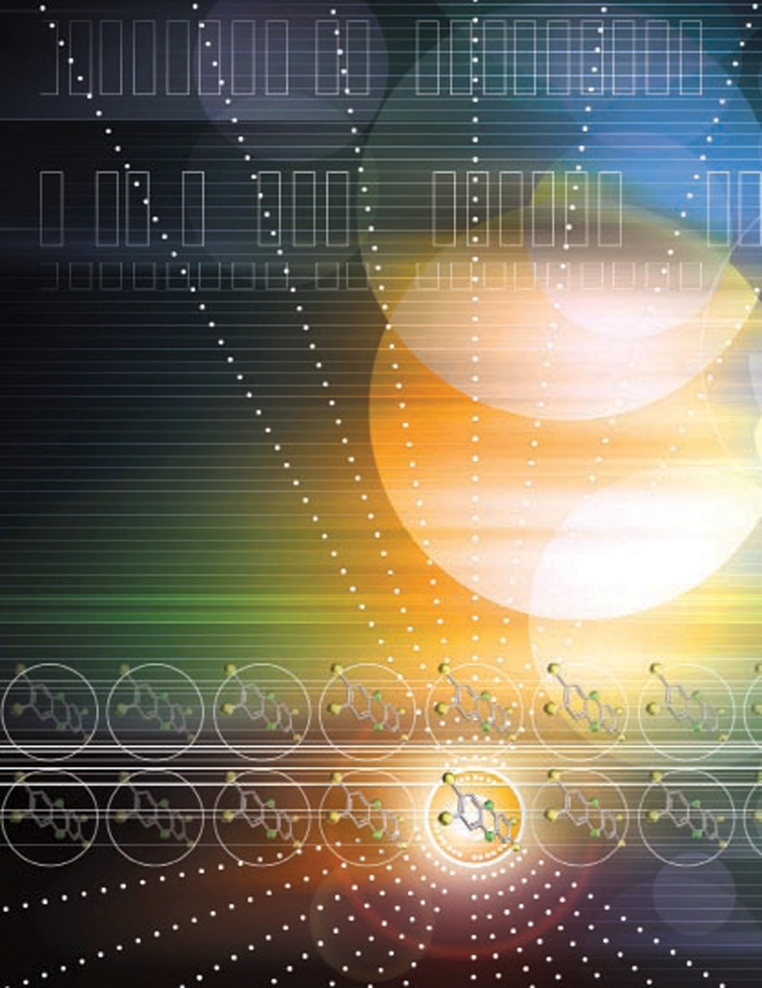# TOX 21: New Dimensions of Toxicity Testing

**DOI:** 10.1289/ehp.117-a348

**Published:** 2009-08

**Authors:** Charles W. Schmidt

**Affiliations:** **Charles W. Schmidt**, MS, of Portland, Maine, has written for *Discover Magazine, Science, and Nature Medicine*. In 2002 he won the National Association of Science Writers’ Science-in-Society Journalism Award

On the ground floor of the National Institutes of Health Chemical Genomics Center (NCGC) in Rockville, Maryland, a $10-million automated laboratory spends all day and night screening chemicals at speeds no team of human researchers could ever match. In a week, depending on the nature of the assay, it can yield up to 2.2 million molecular data points derived from thousands of chemicals tested at 15 concentrations each.

Is this the new face of toxicology? Many experts say the answer could be yes. High-throughput screening tools such as the NCGC’s robotic system—combined with a growing assortment of *in vitro* assays and computational methods—are revealing how chemicals interact with biologic targets. Scientists increasingly believe these tools could generate more accurate assessments of human toxicity risk than those predicted by animal tests now. What’s more, *in vitro* analytical approaches are seen as the best hope for evaluating the enormous back log of untested chemicals in commerce. Estimates vary, but tens of thousands of industrial chemicals are used in consumer products without any knowledge of their potential toxicity. Meanwhile, it takes years and millions of dollars to assess risks for a single chemical using animal testing.

“In almost all aspects, this looks like a paradigm shift in the field,” says John Bucher, associate director of the National Toxicology Program (NTP). “It’s a major change to move from using studies in animals, with which we’re comfortable, to relying mainly on results from biochemical or cell-based assays to make health policy decisions. This is a totally different approach that provides a different kind of information.”

## The Tox21 Partnership

Enabled by new technology, the NTP, the NCGC, and the U.S. Environmental Protection Agency (EPA) are partnering to advance the state of toxicity testing. Specifically, the partners seek to identify new mechanisms of chemical activity in cells, to prioritize the backlog of untested chemicals for more extensive evaluations, and to develop better predictive models of human response to toxicants. Formalized last year in a Memorandum of Understanding, the partnership, dubbed Tox21, responds to a challenge made by the National Research Council (NRC) in its 2007 report *Toxicity Testing in the 21st Century: A Vision and a Strategy*. This report called for transforming toxicology “from a system based on whole-animal testing to one founded primarily on *in vitro* methods that evaluate changes in biologic processes using cells, cell lines, or cellular components, prefer ably of human origin.” In March 2009, the EPA published its own Tox21 agenda, *The U.S. Environmental Protection Agency’s Strategic Plan for Evaluating the Toxicity of Chemicals*, which asserts that “the explosion of new scientific tools in computation al, informational, and molecular sciences offers great promise to . . . strengthen toxicity testing and risk assessment approaches.”

The concept of adding more mechanistic data to risk assessment isn’t new. Before Tox21, physiologically based pharmaco kinetic (PBPK) models, toxicogenomics, and related approaches were already making risk assessment more mechanistically based. But that research didn’t necessarily translate into changes in regulatory policies that govern human exposure, argues Lorenz Rhomberg, a principal with Gradient Corporation, a risk assessment consulting firm in Cambridge, Massachusetts. Despite the availability of mechanistic data, health officials at the EPA have been reluctant to use these data in setting exposure standards because in many cases they would justify higher allowable exposures than those suggested by more conservative default assumptions. Instead, the EPA relies more often on conservative default assumptions about how chemicals affect human beings. “EPA goes by precedent and does things as it did in the past so as to not be arbitrary,” Rhomberg explains. “So, there’s a lot of inertia in the system.”

Robert Kavlock, director of the EPA National Center for Computational Toxicology, says the main difference between Tox21 and prior molecular research in toxicology is one of scale. Scientists have generally focused on hypothesis-driven investigations, such as how a chemical interacts with a specific cell target assumed to play a role in toxicity, he explains. Tox21, on the other hand, relies on unbiased screening methods that don’t assume any prior knowledge about what a chemical might do in the cell. Those investigations ideally will reveal entirely new molecular networks that coordinate toxicity, he says. Kavlock emphasizes that with its new strategy the EPA is demonstrating a willingness to take mechanistic data seriously. “Tox21 was produced with input from senior members across all the EPA offices,” he says. “There’s an explicit recognition that we’re in a scientific transition and that the business part of the agency needs to come along with it.”

## A New Focus on Pathways

Tox21’s essential premise is that scientists can infer human harm from chemicals on the basis of how they activate toxicity path ways in cells. “Toxicity pathway” refers to a chemically induced chain of events that leads to an adverse effect such as tumor formation, explains Raymond Tice, chief of the NTP Biomolecular Screening Branch. Tice emphasizes that these pathways ordinarily coordinate normal processes such as hormone signaling or gene expression. It’s only when they are altered by chemicals or other stressors that harm occurs, he says. “We’re talking about pathways that occur all the time under typical circumstances,” Tice explains. Estrogen-receptor signaling, for instance, is an ordinary feature of nor mal cell biology, “but if it’s inappropriately up- or down-regulated,” Tice says, “it can cause developmental problems.”

Scientists are now attempting to identify and map toxicity pathways and the ways chemicals interact with the biochemical processes involved in cell function, communication, and the ability to adapt to environmental changes. Ideally, these efforts will identify molecular “nodes” vulnerable to chemical exposure. An example of such a node could be a protein that—upon chemical binding—blocks or amplifies estrogen-receptor signaling, altering the pathway’s normal function. This is called a “pathway perturbation.”

After identifying a perturbation, scientists have to put it into a broader context of toxicity in living animals. Doing so requires them to extrapolate a toxic blood or tissue dose from a cell-based response, which can be accomplished with PBPK modeling and computational methods based on human cell circuitry, says Gina Solomon, a senior scientist with the nonprofit Natural Resources Defense Council. Cell-based assays offer some advantages in this respect. Unlike animal tests, which are limited by cost and resource constraints to just a few doses, *in vitro* assays can test chemicals at a broad range of doses that might provide better information about low-dose human effects, scientists say.

The whole process requires a leap of faith that perturbations and associated modeling efforts will accurately predict human effects from chemical exposure, Solomon says. “And this is why risk assessors at EPA have such a hard time with this type of data,” she explains. “It’s not easy to extrapolate from [the results of] a cell-based assay to [exposure effects] in a real population of humans. This is the toughest aspect of pathway-based risk assessment, and it’s one of the main reasons why it’s going to take years for these new approaches to come into widespread use.”

## The Path Forward

Experts anticipate Tox21 will roll out in two phases. In the first, perturbations could guide the selection of chemicals for further testing in animals. With this approach, chemicals that, for instance, trigger oxidative stress (which can lead to inflammation) or impede DNA repair (thus potentially increasing the risk for cancer) could be given high-priority status for testing, whereas those that don’t induce such immediately worri-some effects could be relegated to a lesser concern. The EPA, through its ToxCast™ program, is already exploring how high-throughput systems can be used for prioritization, as is the NTP, in accordance with its own research program for the twenty-first century—the NTP Roadmap that was introduced in 2004.

Kavlock says there’s a crucial need to prioritize chemicals on more of a biological basis. “Right now we’re prioritizing chemicals on the basis of other criteria, such as production volume, the likelihood for human exposure, or their structural similarity to other chemicals with known liabilities,” he says. “By incorporating more biology into prioritization, we think we can do a better job selecting the right chemicals for animal testing. We could also be more efficient in terms of how we conduct these tests.”

In Tox21’s second phase, which some stakeholders say may roll out several decades from now, pathway perturbations could replace animal tests in setting chemical safety standards. Compared with prioritization, this is a far more challenging and elusive goal. Toxicologists have based human standards on the results of animal tests for more than 50 years. Standards for noncarcinogenic chemicals, for instance, are defined by the maximum dose that causes no harm to animals in a toxicity study, divided by numerical factors to reflect data uncertainties. Humans can theoretically tolerate this “reference dose” every day, risk-free, for a lifetime.

Alternatively, carcinogens are regulated with a “cancer slope factor” that scientists extrapolate mathematically from doses that cause tumors in rodents. Tumors often appear only with high doses given for up to two years. Still, EPA regulators cautiously assume dose linearity for carcinogens, meaning even a single molecule of toxicant could, in theory, interact with DNA and cause cancer—in other words, until they can be convinced otherwise, EPA regulators assume there is no dose threshold for carcinogens below which cancer risk is negligible. The cancer slope factor, therefore, aims to limit the number of expected cancers in the exposed population to no more than 1 in 1 million people.

The fact that animal tests rely on doses far higher than those found in the environment raises difficult questions about their relevance to humans. “I’ve spent nearly forty years as a toxicologist trying to relate high-dose animal studies to low-dose human risk,” says Melvin E. Andersen, director of the Program in Chemical Safety Sciences at the nonprofit Hamner Institutes for Health Sciences. “I now believe that’s impossible to do.”

But experts are divided over the degree to which *in vitro* tests can completely replace animals in risk assessment. Andersen’s view—backed by the NRC report, he says—is that testing for perturbations of toxicity pathways, leading to the elimination of animal tests, should be a fundamental goal. “EPA and the NTP want to *in vitro* results to predict high-dose outcomes in animals,” he says. “But that’s backwards—we need to identify cellular targets and then predict what’s going to happen to people at environmentally relevant concentrations. *In vitro* methods will provide better information for such health risk assessment than animal studies. We have to stay current with where modern biology is going. If we don’t, much of what we do in toxicity testing will be regarded as irrelevant.”

Daniel Krewski, director of the R. Samuel McLaughlin Centre for Population Health Risk Assessment at the University of Ottawa and chair of the NRC panel that produced the 2007 report, shares that view. “Let me say this in plain English,” he says. “The thrust of our vision, and also its beauty, is that we will no longer have to regulate on the basis of avoiding what we see in animals but on avoiding perturbations that we see in cell-based tests.”

The EPA approach is more conservative, however, and focuses on prioritizing chemicals for further screening in animals rather than eliminating animals altogether. Kavlock emphasizes that if new technologies help scientists select appropriate chemicals for animal testing, they will go a long way toward making the process more effective and more efficient. “Predicting the future isn’t easy,” Kavlock says. “So, I wouldn’t rule in or rule out that someday we might be able to do [toxicity testing] without animals. But for the foreseeable future, the state of the science just doesn’t allow for that.”

## Overcoming the Status Quo

What animal tests have going for them—apart from a long history in toxicology and a regulatory structure built around their results—is that they integrate responses across physiologic systems. Toxicity is sometimes caused not by a “parent” com pound—the actual chemical to which an animal or human is exposed—but by a metabolite of that compound. Moreover, some chemicals, including some develop mental and neurotoxic compounds, aren’t toxic at the point of exposure but rather at locations elsewhere in the body. John Doull, professor emeritus at the University of Kansas Medical Center, gives the example of chemicals that target certain regions in the brain whose toxic effects are reflected elsewhere, perhaps in terms of gait or vision.

Cell-based assays might not pick up these metabolic or downstream effects, however. A study done in isolated liver hepatocytes, for example, might miss toxicity that occurs only in whole liver, where adjoining cells can metabolize parent chemicals to toxic forms, for instance by what’s known as cytochrome P450-mediated activation.

Christopher Austin, director of the NCGC, concedes metabolic activation poses a tough challenge for *in vitro* research, but not one that can’t be overcome. “This is a very hard problem to deal with,” Austin says. “And we’re approaching it through a major technology development initiative involving co-cultures of hepatocytes and P450-responsive cells. That way, we only see the P450-mediated response if the parent compound is metabolized.”

Another shortcoming with *in vitro* testing is compound integrity. Most laboratories store chemicals in dimethyl sulfoxide (DMSO), a popular solvent that can dissolve both polar (i.e., miscible with water) and nonpolar compounds. But DMSO can also absorb water from the atmosphere and thus degrade the compounds stored in it, explains Adam Yasgar, a research associate at NCGC. “The absorbed water can lead compounds to precipitate, which interferes with the analysis,” he says. “You might not know exactly what you’re testing.”

Yasgar adds that NCGC gets around this problem by testing compounds at many different concentrations. The redundancy of that process leads to more reliable data, he says. But laboratories that rely on single-dose analyses could run into problems, he adds. Kavlock points out that Tox21 plans on chemical characterization of solutions being tested in order to confirm the identity of the chemical, its purity, and its stability in DMSO—an expensive but necessary step, he says, to build confidence in the resulting data.

## The Current Agenda

Tox21 investigators are now conducting proof-of-principle experiments to show that pathway perturbations can predict toxicities already documented in completed animal studies. Their research focuses in part on roughly 10,000 com pounds, including industrial chemicals, pesticide active and inert ingredients, drinking water contaminants, and approved drugs, among others. According to a review in the May 2009 issue of *EHP* by Richard Judson and colleagues, there is at least limited hazard information for about two-thirds of these compounds and detailed toxicology information for about one-quarter of them. The compounds are being screened both at the EPA—through ToxCast—and at NCGC, which is about to purchase yet another robotic laboratory devoted exclusively to Tox21 research. Kavlock says the screens test for a range of end points, such as interactions with nuclear receptors, up-regulation of the p53 tumor suppressor gene, and effects on DNA repair mechanisms.

Meanwhile, scientists are working to identify and map as many toxicity pathways as possible. Just how many pathways might participate in toxicity is a matter of some disagreement, however. Arguing that biology has definable boundaries that are set by the genome, Andersen claims the number is finite. “How many pathways could there be?” he asks. “I don’t know. I’ve suggested, somewhat tongue-in-cheek, that there are exactly 132 of them! The main point is that biology has to be robust, which compels us to believe these pathways are conserved across species [and through evolution]. My personal view is that all toxicity pathways revolve around stress responses and the control of gene expression.” Seen this way, Andersen adds, multiple classes of chemicals could share the same toxicity path ways in spite of differences in their physical structure.

But Katrina Waters, a senior research scientist at Pacific Northwest National Laboratory in Richland, Washington, asserts the number of toxicity pathways might be virtually unlimited. “When you consider the diversity of chemicals facing testing and their potential effects, I don’t think it’s possible to say that some finite number of pathways will predict all adverse events,” she says. “I think it’s probable that each chemical class will have its own set of toxicity pathways for whatever adverse events are characterized for that class.” The debate is far from semantic—the number of toxicity pathways reflects the amount of work ultimately needed to meet the goals of Tox21. For example, Waters explains, if there were only 25 pathways involved in apoptosis, or programmed cell death, scientists could model those pathways mathematically and assume they capture adverse events for every chemical class. “You wouldn’t have to create a new mathematical model for each class; you could simply reuse the same models [and apply them to different chemicals],” she says. “But if you have a limitless number of pathways and conditional interactions between pathways, then you have to repeat the modeling process for every new chemical class [under investigation].”

Going forward, Tox21 offers the opportunity to confer the advantages of high-throughput research on toxicology and risk assessment. But its promise is tempered by the vast research challenges that lie ahead. Scientists are aiming for nothing less than a complete map of the cell circuits that dictate toxicity, assembled from untold millions of data points, converted somehow into some thing useful. Regulatory officials will have to devise ways to replace decisions made on traditional end points with ones made on cell-based findings, Andersen says.

Officials will also have to craft new strategies to explain those findings to the public. “Your average person on the street understands that when something causes birth defects in a rat, that’s something for humans to be concerned about,” says Solomon. “But when you base policies on perturbations of thyroid hormone homeostasis, well, it’s going to be harder for the public to know what to think about that.”

## Figures and Tables

**Figure:**